# Validity and reliability of State-Trait Anxiety Inventory in Danish women aged 45 years and older with abnormal cervical screening results

**DOI:** 10.1186/s12874-020-00982-4

**Published:** 2020-04-23

**Authors:** L. W. Gustafson, P. Gabel, A. Hammer, H. H. Lauridsen, L. K. Petersen, B. Andersen, P. Bor, M. B. Larsen

**Affiliations:** 1 Department of Public Health Programmes, Randers Regional Hospital, Randers, Denmark; 2 Department of Clinical Medicine, Aarhus University, Aarhus, Denmark; 3 Department of Obstetrics and Gynecology, Herning Regional Hospital, Herning, Denmark; 4 Department of Sports Science and Clinical Biomechanics, University of Southern Denmark, Odense, Denmark; 5 Open Patient Data Explorative Network (OPEN) and Department of Obstetrics and Gynecology, Odense University Hospital, Odense, Denmark; 6 Department of Obstetrics and Gynecology, Randers Regional Hospital, Randers, Denmark

**Keywords:** State trait anxiety inventory, Anxiety, Questionnaire, Abnormal cervical screening test, Validation and reliability

## Abstract

**Background:**

State Trait Anxiety Inventory (STAI) scale was developed in the 1980’s and has been widely used both in clinical settings and in research. However the Danish version of STAI has not been validated. The aim of this study was to assess the validity and reliability of STAI - state anxiety scale in Danish women aged 45 years and older with abnormal cervical cancer screening results.

**Methods:**

Women ≥45 years referred with an abnormal cervical cytology and healthy volunteers (*n* = 12) underwent cognitive interview after completing STAI. Further, STAI was sent out in an electronic questionnaire to women (*n* = 109) seen at the gynecological department with abnormal cervical cancer screening test during 2018. Validity and reliability of STAI was evaluated according to the Consensus-based Standards for the selection of health Measurement Instruments (COSMIN) checklist by examining internal consistency, test-retest reliability, measurement error, floor and ceiling, construct validity and content validity.

**Results:**

In the cognitive interviews the content validity was evaluated to be very good. The internal consistency of the scale was excellent with Cronbach’s α = 0.93. Test-retest reliability was good with an intra-class correlation coefficient of 0.80 and the systematic difference between test-retest results was negligible. The construct validity was good.

**Conclusion:**

To our best knowledge, this is the first validation study of the Danish translation of STAI-state anxiety scale. This version of STAI demonstrates an acceptable reliability and validity when used in a gynecological setting.

## Background

Cervical cancer is the fourth most common cancer worldwide with 569,847 new cases and 311,365 deaths in 2018 [1]. Cervical cancer may be prevented by Human Papillomavirus (HPV) vaccination or screening [2]. Screening allows for detection and treatment of cervical precancerous lesion, thereby reducing cervical cancer incidence and mortality. In case of an abnormal screening test, a woman is referred to a gynecologist for colposcopy. Using a colposcope the cervix can be visualized and evaluated, allowing for targeted biopsies of visible lesions. A cone biopsy may subsequently be performed during the diagnostic workup or for treatment of histologically verified lesions. The colposcopic examination carries a low risk of physical harm, such as bleeding and infection, but several studies have demonstrated that an abnormal screening test result and referral for colposcopy may be associated with increased levels of anxiety and discomfort [3–8].

Anxiety among patients in a gynecological setting has been measured using different scales such as the Psychological Consequences Questionnaire, Hospital Anxiety and Depression Scale, the Montgomery-Åsberg Depression Rating Scale, and different versions of State-Trait Anxiety Inventory (STAI) [5, 6, 9]. STAI has the advantage of measuring both trait anxiety, which is considered stable over time, and state anxiety, which is affected by stressful situations such as receiving an abnormal screening result [10, 11].

However, to our best knowledge, the Danish version of STAI has not been validated, neither in a gynecological setting nor in any other Danish setting. In this study we focused on the *state* scale of STAI as this scale has been used to measure anxiety over time and in relation to a given event. We aimed to test validity and reliability of the STAI - state anxiety scale among Danish women aged 45 years and older with abnormal cervical cancer screening results by examining internal consistency, test-retest reliability, measurement error, floor and ceiling, construct validity and content validity.

## Methods

### Setting

The study was conducted at the Department of Obstetrics and Gynecology, Randers Regional Hospital, which is located in Central Denmark Region, one of five Danish regions governing primary and secondary health care services. In Denmark, all citizens have access to free health care due to a tax-financed health care system [12]. Danes are required to communicate with health authorities and hospitals through secure digital mail which less than 10% of the Danish citizens are exempt from [13]. This digital mail allows for communication of digital questionnaires directly to Danish citizens.

### Study design

A two-step validation study of the Danish version of STAI – state anxiety scale was conducted to evaluate how well the Danish translation of the scale performed in a gynecological outpatient clinic. Cognitive interviews were conducted to test content validity and a questionnaire study was conducted to test reliability, floor and ceiling effect, and construct validity.

### Cognitive interviews

#### Study population

We conducted individual interviews with women aged 45 years and older with and without an abnormal cervical screening test. Women were eligible for interview if they were referred to the Department of Obstetrics and Gynecology, Randers Regional Hospital or to a private gynecologist in Randers due to an abnormal screening test. Women were considered ineligible if they were unable to speak and understand Danish. A convenience sample of women without an abnormal screening result was also interviewed.

#### Data collection

Individual interviews were conducted from 4th December 2018 to 13th December 2018. Eligible women were asked by the medical staff at the gynecological department or at the private gynecologist if they were interested in participating. Patients agreeing to participate were instructed to fill out the questionnaire in a quiet room. Subsequently, they were interviewed by author LWG who is trained in qualitative research. A semi-structured interview guide was designed to cover the women’s opinion in terms of comprehensiveness, comprehensibility, and relevance of the questionnaire items. Each interview was documented by taking notes during the interview. Interviews were conducted until data saturation was reached.

#### Analyses

Open-ended questions were asked about the following categories: understanding of the answer categories of the scales, time-consumption, layout of the questionnaire, the comprehensibility and relevance of the questions, and how they interpreted the questions. An inductive analysis was performed to make more general conclusions on the respondents’ statements.

### Questionnaire study

#### Study population

**Fig. 1:**
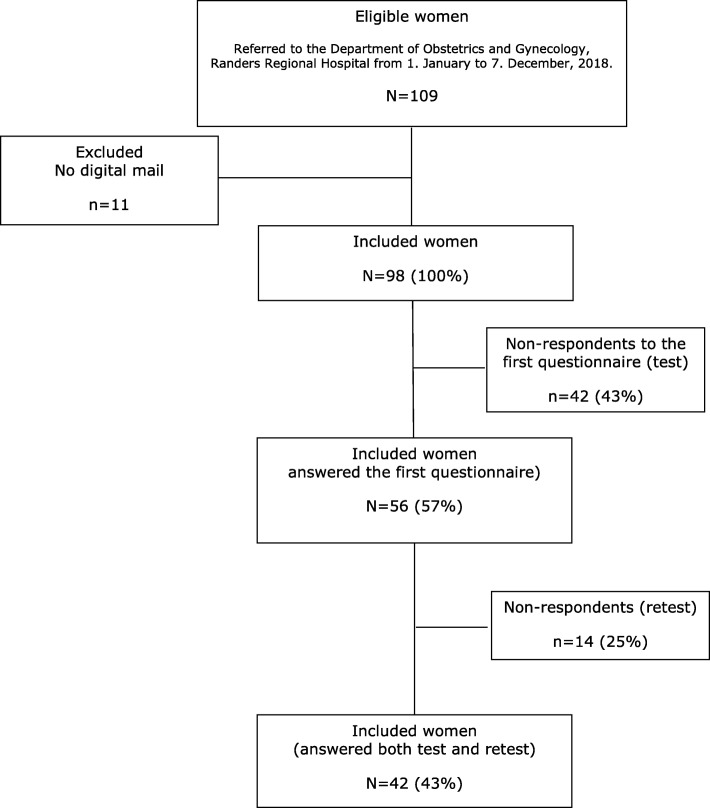
Flow chart of women included in the quantitative validation of STAI-state anxiety scale

The questionnaire was sent to women 45 years or older who had been referred to the Department of Obstetrics and Gynecology, Randers Regional Hospital from 1 January 2018–7 December 2018 due to an abnormal screening result. Women exempted from the digital mail were excluded (Fig. 1).

#### Data collection

The questionnaire consisted of the Danish translation of STAI - state anxiety scale along with Short Form 12 Health Survey (SF-12) which was used for hypothesis testing to assess construct validity of STAI - state anxiety scale.

It was set up in REDCap™ (Research Electronic Data Capture) hosted at Aarhus University. REDCap is a secure, web-based software platform designed to support data capture for research studies [14, 15]. All questions in the questionnaire were mandatory to ensure no missing values.

The questionnaire was sent electronically through the digital mail to eligible women on 3rd January 2019 along with information on the validation process of the questionnaire, including the reason for test-retest, and an individual link to the electronic questionnaire. One week later a reminder was sent to non-responders. Fourteen days after the first questionnaire was answered the same questionnaire was sent for a test-retest and 1 week later a reminder was sent to non-responders in the retest.

#### Questionnaires

##### STAI - state anxiety scale

The Danish version of STAI - state anxiety scale was provided by the copyright owner (Mind Garden, Inc., USA) with no information on the translation process or validation data.

STAI - state anxiety scale consists of 20 items with response options based on a self-reported 4-point Likert scale (“Not at all”, “Somewhat”, “Moderately so” and “Very much so”). The state anxiety score ranges from a minimum of 20 to a maximum of 80. A low score indicates no or little anxiety while a higher score indicates a higher level of anxiety. All items of the state anxiety scale were found to belong to one uni-dimensional scale [16].

Norm data for STAI - state anxiety scale for working adults based on a total of 1838 employees of the Federal Aviation Administration (1387 males; 451 females) was available from the STAI Manual. Although most were non-Hispanic white, the sample was heterogeneous with regard to educational level and age. The mean score for working females aged 40–49 was 36.03 (SD 11.07) and 32.20 (SD 8.67) for working females aged 50–69 years [11].

##### Short form 12 health survey (SF-12)

SF-12 is a health-related quality of life questionnaire consisting of 12 items divided into two sub-scales of physical and mental health. The total score ranges from 0 to 100, where zero indicates the lowest level of health-related quality of life and 100 indicates the highest level of health-related quality of life.

### Analyses

Validity and reliability of the scale was evaluated according to the COSMIN (COnsensus based Standards for the selection of health status Measurement INstruments) checklist [17]. All statistical analyses were conducted on the initial test results and only measurement error and test-retest reliability included results from the retest.

#### Reliability

##### Internal consistency

Internal consistency was assessed by Cronbach’s α which was presented for the scale as a whole with 95% confidence interval (CI). Cronbach’s α values between 0.7–0.9 were regarded as acceptable [18].

##### Test-retest reliability

The test-retest reliability was assessed by calculating the intra-class correlation coefficient (ICC) using the two-way mixed-effect model with interaction for the absolute agreement between single scores [19]. ICC between 0.5–0.75 indicates moderate reliability, ≤0.75–0.9 indicates good reliability and greater than 0.9 indicates excellent reliability [20].

##### Measurement error

Measurement error was presented as the limits of agreement with the test-retest score, and differences were plotted against the average test-retest scores in a Bland-Altman plot [21]. Furthermore, we calculated the smallest detectable change (SDC) defined as 1.96 x SD_difference_. This equals the limits of agreement without the systematic error [22].

##### Floor and ceiling effect

Floor and ceiling effects were illustrated in histograms for each item and the total score, and assessed as the number of participants achieving the highest or lowest possible score. If more than 15% scored at either end of the scale, we defined this as a floor/ceiling effect [23].

##### Construct validity

Construct validity was assessed by performing hypothesis testing against SF-12 scores in the questionnaire using a Spearman’s rank correlation. Correlation coefficients above > 0.5 indicated a strong correlation, 0.3–0.5 indicated a moderate correlation and < 0.3 indicated a weak correlation [24]. Further, hypothesis testing was based on the mean score for American working females aged 50–69 years provided in the STAI state manual [11] using the Student’s t-test.

The first hypothesis was that there would be a strong correlation (≥ 0.5) between anxiety measured by the STAI-state anxiety scale and mental health measured by SF-12, as the two scales measure similar constructs. Since higher STAI-state anxiety scores indicate higher anxiety and lower SF-12 scores indicate poor mental health, the association was expected to be negative. Further, it was hypothesized that there would be a moderate (0.3–0.5) and negative correlation between high levels of anxiety and poor physical health as high levels of anxiety do not necessarily affect one’s physical health. The third hypothesis was that the mean scores reported in the STAI manual (women 50–69 years) would not be statistically significant different from the Danish STAI-state anxiety scale mean scores (*p* > 0.05).

All statistical analyses were conducted in Stata/SE 15 (StataCorp. 2017. Stata Statistical Software: Release 15. College Station, TX: StataCorp LLC).

### Study approvals

According to EU’s General Data Protection Regulation (article 30), the project was listed at the record of processing activities for research projects in Central Denmark Region (J. No. 1–16–02-528-18). The present study has been presented to the Central Denmark Region Committees on Health Research Ethics. The Committee decided that according to the Danish Act on Research Ethics Review of Health Research Projects (Act number 111/2017), this study should not be notified to the Committees (j.no:1–10–72-4-17).

## Results

### Cognitive interviews

Seven women with abnormal cervical screening results (mean age 63.9 years (SD 7.99)) and five women without abnormal cervical screening results (mean age 51.8 years (SD 8.98)) were included for individual interviews.

#### Content validity

The 12 women both with and without abnormal cervical screening results reported that the STAI- state anxiety scale was relevant and easy to interpret. They liked that it was short and yet comprehensive. They found the response options to be appropriate and the wording was easy to understand. They did not suggest adding questions or changing the wording of the existing questions. From the individual interviews, content validity was evaluated to be very good.

### Questionnaire study

#### Participants

A total of 109 women were eligible for validation of the reliability and validity of STAI-state anxiety scale. Of these, 98 women had electronic mail and were invited to participate (mean age 58.1 years (SD 9.12)). A total of 56 women completed the first questionnaire (57%) (mean age 59.3 years (SD 9.38)), and a total of 42 women completed both test and retest questionnaires (43%) (mean age 57.9 (SD 8.22)) (Fig. 1).

The overall mean score of STAI – state anxiety scale was 32.6 (SD 10.4). The mean score for women aged 45–49 years was 32.6 (SD 11.1), for women aged 50–69 years it was 32.3 (SD 10.6) and for women 70 years or older it was 34.4 (SD 10.41).

#### Reliability

##### Internal consistency

The internal consistency of the scale was excellent (Cronbach’s α = 0.93; 95% CI: 0.91; 0.97).

##### Test-retest reliability

The test-retest reliability was good with an ICC of 0.80 (95% CI: 0.66; 0.89).

##### Measurement error

**Fig. 2:**
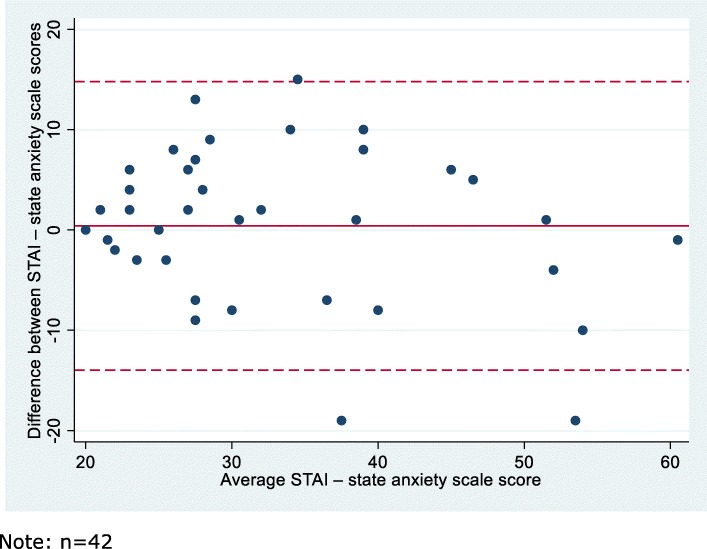
Bland-Altman plot of test-retest score differences against mean test-retest scores with 95% limits of agreement (*n* = 42)

The systematic difference between test and retest results was 0.40 points (95% CI: − 0.88; 2.69), and the lower and upper limits of agreement was − 13.98 and 14.79, respectively (Fig. 2). The SDC was 14.4 points which means that change outside 14.4 points can be considered a true change [18].

##### Floor and ceiling effect

**Fig. 3:**
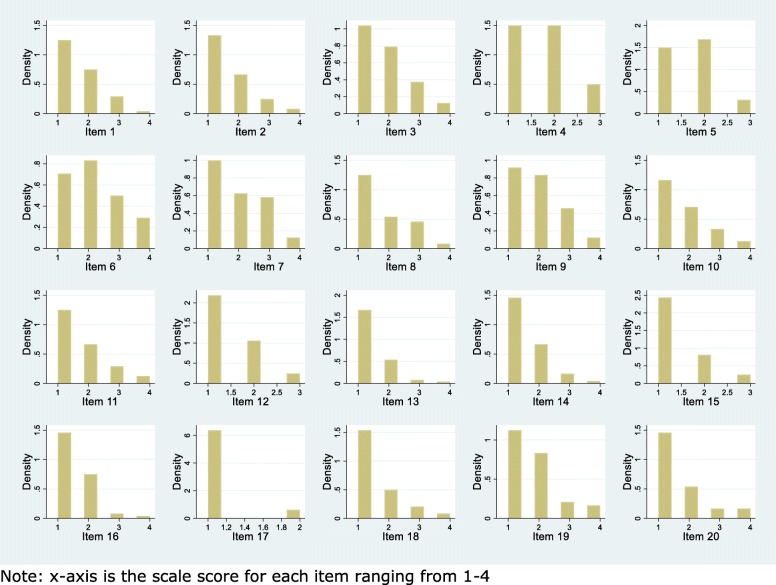
Histograms of item score for each item in the STAI-state anxiety scale (*n* = 56)

**Fig. 4:**
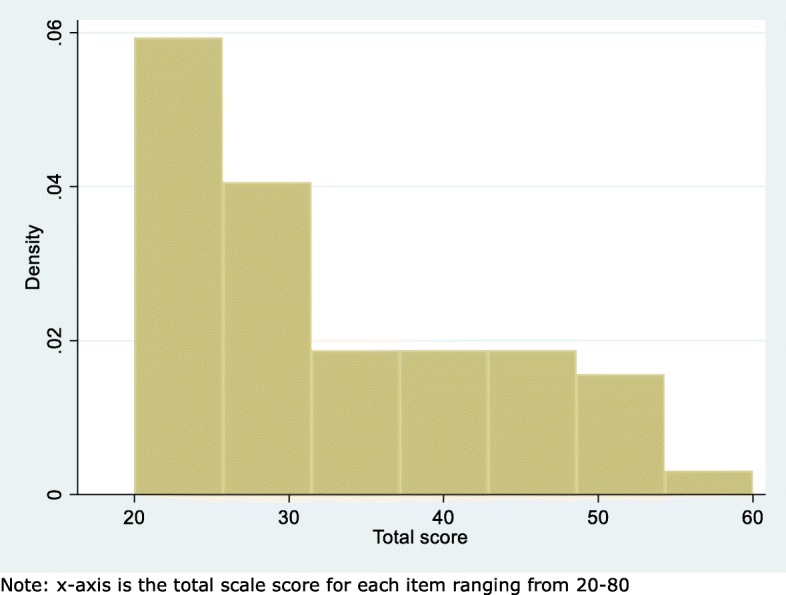
Histogram of total score of the STAI-state anxiety scale (*n* = 56)

In most items, all possible answers had been used, with a tendency towards lower scores in all items (Fig. 3). Total score ranged from 20 to 60 out of 80 possible, with lower scores being most common (Fig. 4). Five (8.9%) participants had a total score of 20 and 0 had a total score of 80 revealing no floor or ceiling effects.

### Construct validity

#### Hypothesis testing

Correlation between the STAI state anxiety scale and SF-12 physical health was negative and moderate (− 0.3082, 95% CI: − 0.5610; − 0.0555) and for SF-12 mental health negative and strong (− 0.6752, 95% CI: − 0.8043; − 0.5461) both confirming our hypotheses (Table 1).

**Table 1:** Hypothesis testing against SF-12 mental and psychical health and mean scores in the STAI manual

Hypothesis testing	Correlation	Difference (50–69 yrs)
SF-12 mental health scale versus STAI - state anxiety scale*	Spearman’s rho = − 0.6752 95% CI (− 0.8043; − 0.5461)	
SF-12 physical health scale versus STAI state anxiety scale**	Spearman’s rho = − 0.3082 95% CI (− 0.5610; − 0.0555)	
STAI state anxiety scale (manual) versus STAI state anxiety scale***		Difference = 0.14 95% CI (−2.82;3.10)

* Scales are not measuring the same construct. The correlation is expected to be negative and weak to moderate (− 0.3;-0.5) ** Scales are not measuring the same construct. The correlation is expected to be negative and strong ≥0.5. *** Scales are measuring the same construct and are expected to have a *p* value > 0.05

There was a difference of 0.14 (95% CI: − 2.82; 3.10) in the mean score for STAI state anxiety scale ranging from 20 to 80 between the STAI manual and our findings confirming our hypothesis of no difference (Table 1).

## Discussion

### Main findings

To our best knowledge, this is the first validation study of the Danish translation of STAI-state anxiety scale. The results indicated that the scale holds acceptable validity in a population of Danish women older than 45 years in a gynecological setting. Content validity, internal consistency, test-retest reliability and construct validity was good. The mean score resembled norm data for working females, indicating that women with an abnormal cervical screening test within the last year are fairly unaffected by anxiety and mimics the background population.

### Strengths and limitations

This study benefitted from the two-step validation procedure, including both interviews and questionnaire. Even though Mind Garden, Inc., USA, provides no information on the translation process or validation data on the Danish version of the STAI-state anxiety scale, the individual interviews confirmed that Danish women with and without an abnormal screening test found the scale comprehensible and relevant. This may indicate that both the original concept of STAI-state anxiety and the Danish translation is accepted among Danish women.

The main limitation of the study was the relatively low number of women included in the questionnaire study. However, we included all women referred to the Department of Obstetrics and Gynecology, Randers Regional Hospital from 1 January 2018–7 December 2018 who had an abnormal screening test. Due to the relatively small study population we were unable to conduct a factor analysis to confirm that the scale was unidimensional, and we therefore had to rely on the validation of the original scale.

Furthermore, we cannot rule out selection bias, as only 57% responded (56/98) in the initial questionnaire, and 43% (42/98) in the retest, and because we only included women with digital secure mail. We were unable to determine the magnitude of this potential bias, as we only had information on age of non-respondents and because we have no information on women exempt from digital mail. However, respondents had similar age distribution as those who did not respond. Still, the respondents may be a selected group in terms of health status, socioeconomic status, and other unknown factors. This may partly explain that no one scores at the highest end of the scale if those most affected by anxiety did not participate in this study, which is always a dilemma in research. Further, the women were recruited based on a visit to a gynecologist within the past year which may further explain that the mean score resembled the mean score from norm data.

Our results showed a relatively high SDC for several reasons. Recruiting women at the day of their gynecology appointment and letting them fill out the questionnaire on that day might have yielded higher scores which could not be reproduced in a retest after 2 weeks, because STAI - state anxiety scale measures transitory anxiety. Furthermore, we did not include a stability anchor to exclude those who had changed over the 2 weeks between test and retest, and this probably resulted in an inflated measurement error. Consequently, our SDC is probably too high and therefore has to be used and interpreted with caution.

Another limitation was that the digital questionnaire was set up not to allow missing values which would normally be avoided in validation studies. However, since item formulation, relevance and acceptability were already tested in the interviews, it was decided to help the respondents not to miss any questions due to the digital setup where it may be difficult to visualize if all answers had been marked.

### Discussion of results and comparison to other studies

Even though STAI-state anxiety scale has been widely used for decades, we were only able to find few other validation studies, including a Greek [25], Japanese [26], Taiwan [27] and a Malaysian study [28]. The majority of these studies was published before the COSMIN checklist and varies widely in design and methods.

None of the previous studies conducted confirmatory factor analysis to confirm uni-dimensionality of the state anxiety scale. Thus, factor analysis is only reported in the STAI manual. In previous studies, a high internal consistency was found (0.87–0.93) [25–28], which is in line with our findings.

Our results are in line with a Swedish cohort study [5], where they measured STAI state anxiety in women referred for colposcopy. They found a mean STAI state anxiety score at the first colposcopy visit to be 42.7, declining to 35.0 after 2 years follow-up. A reference group with healthy women participating in the cervical cancer screening program was found to have a mean state anxiety score at 34.8. Similar results were seen in the study from J. Byrom et al., in which women had a mean score of 45.94 before colposcopy and 36.91 6 weeks after colposcopy [3]. In a Finnish study, women referred for colposcopy had a mean score of 34 both at baseline, six and 12 months after referral to colposcopy [4] These numbers are similar to our results and are measured in a comparable target population.

## Conclusion

The Danish version of STAI-state anxiety scale demonstrates an acceptable reliability and validity when used in a gynecological setting. The most important issue for future studies is the considerable measurement error related to the scale. Thus, we recommend using a stability anchor along with the STAI-state anxiety scale.

## Data Availability

The dataset generated and analyzed in this study are not available for the public due to Danish legislation. Data are available on request for researchers who meet the criteria for access to patient’s confidential data.

## Abbreviations

STAI: State-Trait Anxiety Inventory
COSMIN: Consensus-based Standards for the selection of health Measurement Instruments
HPV: Human Papillomavirus
SF-12: Short form 12
CI: Confidence interval
SD: Standard deviation
REDCAP: Research Electronic Data Capture
ICC: intra-class correlation coefficient
SDC: Smallest Detectable Change
